# Lutathera^®^ Orphans: State of the Art and Future Application of Radioligand Therapy with ^177^Lu-DOTATATE

**DOI:** 10.3390/pharmaceutics15041110

**Published:** 2023-03-31

**Authors:** Luca Urso, Alberto Nieri, Licia Uccelli, Angelo Castello, Paolo Artioli, Corrado Cittanti, Maria Cristina Marzola, Luigia Florimonte, Massimo Castellani, Sergio Bissoli, Francesca Porto, Alessandra Boschi, Laura Evangelista, Mirco Bartolomei

**Affiliations:** 1 Department of Translational Medicine, University of Ferrara, Via Aldo Moro 8, 44124 Ferrara, Italy; rsulcu@unife.it (L.U.); ctc@unife.it (C.C.); francesca.porto@edu.unife.it (F.P.); 2 Department of Nuclear Medicine, PET/CT Centre, S. Maria della Misericordia Hospital, 45100 Rovigo, Italy; mariacristina.marzola@aulss5.veneto.it; 3 Nuclear Medicine Unit, Oncological Medical and Specialist Department, University Hospital of Ferrara, 44124 Cona, Italy; a.nieri@ospfe.it (A.N.); m.bartolomei@ospfe.it (M.B.); 4 Nuclear Medicine Unit, Fondazione IRCCS Ca’ Granda, Ospedale Maggiore Policlinico, 20122 Milan, Italy; angelo.castello@policlinico.mi.it (A.C.); luigia.florimonte@policlinico.mi.it (L.F.); massimo.castellani@policlinico.mi.it (M.C.); 5 Nuclear Medicine Unit, AULSS1 Dolomiti, San Martino Hospital, 32100 Belluno, Italy; paolo.artioli@aulss1.veneto.it (P.A.); sergio.bissoli@aulss1.veneto.it (S.B.); 6 Department of Chemical, Pharmaceutical and Agricultural Sciences, University of Ferrara, 44121 Ferrara, Italy; alessandra.boschi@unife.it; 7 Department of Medicine DIMED, University of Padua, 35128 Padua, Italy; laura.evangelista@unipd.it

**Keywords:** Lutathera^®^, ^177^Lu-DOTATATE, thera(g)nostics, radioligand therapy (RLT), peptide receptor radionuclide therapy (PRRT), somatostatin receptor (SSTR), neuroendocrine tumors (NET), pheochromocytoma and paraganglioma, meningioma, bronchial carcinoid

## Abstract

Lutathera^®^ is the first EMA- and FDA-approved radiopharmaceutical for radioligand therapy (RLT). Currently, on the legacy of the NETTER1 trial, only adult patients with progressive unresectable somatostatin receptor (SSTR) positive gastroenteropancreatic (GEP) neuroendocrine neoplasms (NET) can be treated with Lutathera^®^. Conversely, patients with SSTR-positive disease arising from outside the gastroenteric region do not currently have access to Lutathera^®^ treatment despite several papers in the literature reporting the effectiveness and safety of RLT in these settings. Moreover, patients with well-differentiated G3 GEP-NET are also still “Lutathera orphans”, and retreatment with RLT in patients with disease relapse is currently not approved. The aim of this critical review is to summarize current literature evidence assessing the role of Lutathera^®^ outside the approved indications. Moreover, ongoing clinical trials evaluating new possible applications of Lutathera^®^ will be considered and discussed to provide an updated picture of future investigations.

## 1. Introduction

Radioligand therapy (RLT) with radiolabeled somatostatin analogues (SSA) is currently a mainstay in advanced gastroenteropancreatic (GEP) neuroendocrine tumor (NET) treatment, as it represents an ideal model of a modern system of personalized medicine. However, to reach this achievement required quite a long and challenging scientific journey. The first experiences with radiolabeled SSA, dating back to the late 1990s, employed yttrium-90 labelled SSA as radiopharmaceutical for RLT [1,2]. However, renal toxicities were not negligible, hindering the widespread of this therapeutic option [3]. Afterwards, the attention shifted to lutetium-177 labelled SSA, due to their more favorable toxicity profile and to the first positive experiences obtained with [^177^Lu][Lu-DOTA,Tyr3]octreotate ([^177^Lu]Lu-DOTATATE) [4]. The growing interest in this radiopharmaceutical led to the NETTER-1 study, a phase III multicenter trial whose results started a new era, demonstrating the most favorable outcomes of [^177^Lu]Lu-DOTATATE RLT plus octreotide LAR 30 mg versus octreotide LAR 60 mg alone [5]. As a consequence of NETTER-1 results, [^177^Lu]Lu-DOTATATE was finally approved by the European Medicines Agency (EMA) in September 2017, the Food and Drug Administration (FDA) in January 2018, the Canada Health in January 2019, and the State of Israel Ministry of Health in July 2019 (Lutathera^®^) [6,7,8,9,10]. Currently, Lutathera^®^ is approved in 23 countries worldwide. However, this should be considered only a partial achievement as a large portion of tumors overexpressing somatostatin receptors (SSTR) still cannot be treated with Lutathera^®^, giving rise to the so-called “Lutathera Orphans”. Indeed, Lutathera^®^ is currently administered in a protected hospitalization regime and is indicated in adult patients diagnosed with well-differentiated (G1 and G2) gastroenteropancreatic neuroendocrine tumors (GEP-NET) that are progressive, non-removable or metastatic, and positive to the receptors for somatostatin. [11]. Therefore, paediatric patients cannot be treated with Lutathera^®^. Similarly, patients with newly diagnosed or stable metastatic disease, even if symptomatic or affected by a large burden of disease, are not eligible for this therapy despite promising literature evidences [12]. Finally, G3 NET, neuroendocrine carcinomas (NEC), or extra-GEP-NET patients are still Lutathera^®^ orphans as well, even though they often show intense overexpression of SSTRs at functional imaging. Among extra-GEP-NET group, bronchial carcinoids, pheochromocytomas (PHEOs), paragangliomas (PGLs) and neuroblastomas, meningiomas, unknown primary (CUP) NETs and some other infrequent tumors can all be listed.

The aim of this review is to provide an updated picture of the literature’s reporting of [^177^Lu]Lu-DOTATATE treatment outside current indications. Moreover, ongoing clinical trials aiming to evaluate new possible applications of Lutathera^®^ will be considered and discussed in order to provide a starting point for allowing access to RLT for a greater percentage of NET patients in the future.

## 2. Materials and Methods

A critical literature review was conducted on PUBMED and Scopus databases up to the 31 January 2023 with the aim of analyzing papers describing the applications of Lutathera^®^ or [^177^Lu]Lu-DOTATATE outside currently approved indications. In particular, evidence discussing RLT in G3 GEP-NETs, bronchial carcinoids, pheochromocytomas and paragangliomas, meningiomas, CUP-NETs, and other rare tumors overexpressing SSRT2 and RLT retreatment (named salvage RLT) were all collected and discussed. Only articles in the English language were selected. Papers reporting the use of RLT associated with other oncological therapies were not considered.

A research of ongoing clinical trials was performed on https://clinicaltrials.gov/ (accessed on 13 February 2023) [13] using the following keywords: Lu-177-DOTA-octreotide; Lu-177 DOTATATE; ^177^Lu-DOTA-octreotate; LUTETIUM OXODOTREOTIDE LU-177; Lutetium Lu 177-DOTA-Tyr3-Octreotate; ^177^Lu-DOTA-Tyr3-Octreotate; ^177^Lu-DOTA0-Tyr3-Octreotate; Lu-DOTA-TATE; lutetium Lu 177-DOTATATE.

## 3. Results

### 3.1. G3 GEP-NET and NEC

Currently, Lutathera^®^ is approved only for G1 and G2 GEP-NETs. Only a few examples, mostly retrospective studies, have been published regarding the possible role of RLT in G3 GEP-NET or NEC [14,15,16,17]. Overall, results have demonstrated a survival benefit, expressed as improved progression-free survival (PFS) (between 9 and 23 months) and overall survival (OS) (between 19 and 53 months) as well as a significant disease control rate (DCR) ranging between 30% and 80%. In particular, patients with a Ki-67 index < 55% seem to better respond to RLT in comparison to those with a higher proliferation index. Furthermore, the rates of toxicity in G3 patients were only mild and comparable to those involving G1–G2 patients [18]. Therefore, following these preliminary encouraging results, ESMO guidelines suggest RLT as a therapeutic option for G3 NET patients, although with a level of evidence of IV and grade of recommendation of C [19].

Based on valid results of the above mentioned retrospective studies, two phase III clinical trials are currently ongoing: the NETTER-2 (NCT03972488) and the COMPOSE (NCT04919226) [20,21]. The first is a multicenter, randomized, and open-label study aiming to assess the impact of Lutathera^®^ combined with long active octeotride on PFS. In particular, the study is enrolling GEP-NET patients with G2 (Ki-67 ≥ 10%) and G3 (Ki-67 ≤ 55%) diseases. Conversely, the COMPOSE trial aims to evaluate the outcomes and safety of RLT with another radiopharmaceutical agent, [^177^Lu]-[LuDOTA,Tyr3]octreotide ([^177^Lu]Lu-DOTATOC), compared to chemotherapy as a standard of care in patients with G2-G3 GEP-NET and a Ki-67% comprised between 15% and 55%. Unlike the NETTER-2, COMPOSE trial requires 2-deoxy-2-[^18^F]fluoro-d-glucose ([^18^F]FDG) PET/CT imaging as inclusion criteria, following several reports in literature highlighting its growing importance [22,23,24,25]. Indeed, a deep discordance between [^18^F]FDG and [^68^Ga]Ga-DOTA-SSTR imaging might limit RLT efficacy, allowing the consideration of other treatments. As a matter of fact, a recent EANM Focus 3 consensus suggests the routine employment of [^18^F]FDG PET/CT for all G3 NET candidates for RLT, as high-proliferation tumors are most likely to show mismatched [^18^F]FDG positive and [^68^Ga]Ga-DOTA-SSTR negative lesions [26].

### 3.2. Bronchial Carcinoids

Bronchial carcinoids (BCs) are uncommon neuroepithelial neoplasms representing a separate biological–molecular entity from neuroendocrine lung carcinomas [27]. BCs are classified as typical carcinoids (TCs)—characterized by slow growth and better outcomes—and atypical carcinoids (ACs)—very infrequent (about 0.2%), aggressive tumors with great potential of metastatic spread. Differently from GEP-NET, Ki-67 expression does not differentiate between TCs and ACs, and according to WHO guidelines, BC grading relies on mitotic index and tumor necrosis [28].

Treatment of bronchial carcinoids is not simple and requires a multidisciplinary approach. Surgery remains the mainstay of treatment for local disease, while in advanced disease, management includes chemotherapy, “cold” somatostatin analogues, immunotherapy, everolimus, and others target therapies [28,29]. RLT is an option for selected patients with advanced or metastatic BCs overexpressing SSTR in progression to “cold” SSAs therapy. Although the experience of [^177^Lu]Lu-DOTATATE is more limited in BCs than GEP-NET, off-label use can be considered with promising results [30,31]. A prospective phase II trial in 34 patients with stage IV BCs treated with cumulative activity of 18.5–27.8 GBq in four or five cycles of [^177^Lu]Lu-DOTATATE documented a DCR of 80% (6% complete response, 27% partial response, and 47% stable disease) and a median PFS of 20 months [32]. In this study, negative prognostic factors were AC histology, tumor thyroid transcription factor-1 (TTF-1) expression, and a positive [^18^F]FDG PET imaging. Comparable results (median PFS of 17 months) were reported in a group of patients with diffuse extrahepatic metastases treated with RLT after several lines of therapies [33].

Higher activities were administered in a study by van Essen et al. [34], who treated nine patients with metastatic BCs, delivering a cumulative dose of 22.2–29.6 GBq of [^177^Lu]Lu-DOTATATE, demonstrating an overall response rate comparable to that of other GEP-NET (50% vs. 47%, respectively for BCs and GEP-NET). Moreover, tumor regression was reported in 66.6% of patients, without any outcome discrepancies between TCs and ACs. Comparable median OS and response rate between BCs and others GEP-NETs were reported also in another recent retrospective study [35]. Likewise, the efficacy of RLT in BCs was demonstrated by Brabander et al. [36] in a study involving 443 patients affected by midgut, bronchial, and CUP-NETs treated with 7.4 GBq of [^177^Lu]Lu-DOTATATE every 8 weeks. The authors found that objective response rate (ORR) for BCs was 30%, and an additional 30% of patients had a stable disease. Nevertheless, median OS for BCs was 52 months versus 71 months for pancreatic-NET.

Functioning BCs could secrete various hormones, causing ectopic Cushing syndrome, syndrome of inappropriate antidiuretic hormone secretion (SIADH), and carcinoid syndrome. A recent study [37] analyzed the effect of [^177^Lu]Lu-DOTATATE treatment in patients with NETs and carcinoid syndrome insufficiently controlled by “cold” SSa, demonstrating that RLT can be effective in reducing diarrhea and flushing, and it can be considered as an alternative treatment in symptomatic patients. RLT has also been demonstrated to improve the quality of life in BCs and in GEP-NETs [38].

Long term results were analyzed by Mariniello et al. [39] in 114 patients with advanced BCs treated with different RLT protocols. They documented a median PFS and OS of 28.0 and 58.8 months, respectively, while morphological responses were observed in 26.5% of patients. [^177^Lu]Lu-DOTATATE monotherapy protocol resulted in the highest 5-year OS (61.4%), despite tandem protocol ([^90^Y]Y-DOTATOC + [^177^Lu]Lu-DOTATATE) provided the highest response rates (38.1%). Best outcome was reached in patients who underwent RLT in early stage of disease, suggesting that this treatment should also be considered for newly diagnosed unresectable BCs. Moreover, despite most patients having only mild and transient adverse events, patients with hematologic toxicity showed worse survival outcomes.

### 3.3. Pheochromocytomas and Paragangliomas

PHEOs and paragangliomas PGLs (collectively named PPGLs) are infrequent neuroendocrine neoplasms originating from chromaffin cells. PHEOs arise from the adrenal medulla, whilst PGLs are extra-adrenal tumors, potentially arising from any part of the sympathetic or parasympathetic nervous system [40,41]. The majority of these neoplasms are considered benign even though the local growth can cause mass effects, particularly for PGLs localized in the head and neck region [42]. Nevertheless, a variable number of these tumors, ranging between 2–26%, can develop metastasis. Currently, histopathology is unable to distinguish between benign and malignant PHEOs or PGLs. Therefore, most updated guidelines suggest considering that any lesion can have metastatic potential [43,44]. A strong genetic component is described in these neoplasms, and the most frequent mutations involve succinate dehydrogenase (comprehending A/B/C/D/AF2, collectively called SDHx mutations) and von Hippel-Lindau (VHL) genes. In particular, patients with SDHB mutation—which is often inherited as an autosomal dominant germline—have a high risk of metastatic disease occurrence [45]. Overall, PGLs originating from the sympathetic chain and PHEOs can be functioning, as they may secrete catecholamines. The deriving sympathetic overactivity can cause secondary hypertension, palpitations, headache, sweating, and a state of anxiety. Therefore, the therapeutic management of these patients is often complicated as a balanced alpha- and beta-adrenergic blockade is needed [41].

Metastatic PPGLs represent a challenging disease entity with limited therapeutic options available [46]. A nuclear medicine theranostic approach utilizing [^123^I]metaiodobenzylguanidine ([^123^I]MIBG) as diagnostic and [^131^I]MIBG as therapeutic agent has been tested and approved by FDA (Azedra^®^) [47]. However, this theranostic model presents several drawbacks, including dosimetric issues and consequent high risk of therapy-related myelosuppression [46]. In recent years, growing evidence of the overexpression of SSTR type 2 and 3 in PPGLs determined an increased use of [^68^Ga]Ga-DOTA-SSA PET/CT [48,49,50]. Overall, a lesion-based sensitivity of 92% was reported across a wide range of mutations, outperforming any other functional imaging investigation, in particular for SDHx mutated metastatic PPGLs and in non-metastatic head and neck PGL [49].

As a consequence, we saw a rising interest in a potential radiolabeled-SST agonist theranostic approach for PPGLs, with several spontaneous studies reporting of the treatment of metastatic or inoperable tumors with Lutathera^®^ and other similar radiocompounds, with promising preliminary results [51,52]. The first RLT experience in PPGLs was reported by van Essen et al. [53], who treated a heterogeneous cohort of patients, including 12 PGLs. Despite the authors reporting lower response rates than those obtained in GEP-NET patients, ORR and DCR were 18% and 73%, respectively. These results are consistent with other reports in the literature [54,55,56,57]. Kong et al. [41] treated 20 PPGLs (8 PHEOs and 12 PGLs) with [^117^Lu]Lu-DOTATATE, 14 due to uncontrolled secondary hypertension and 6 to radiological PD. A DCR equal to 86% was reached, including five patients with ORR (36%). Of note, 62% of symptomatic patients required a dose reduction of antihypertensive medications, and 57% reported a subjective therapeutic benefit in terms of tumor-related symptoms. Comprehensive PFS was 39 months, including two patients with early recurrence (2 and 5 months post-RLT, respectively) and one patient with remarkable tumor downsizing that allowed a second-step curative liver surgery. Of note, the patient was still disease-free at the time of the study. The results of the largest cohort of PPGLs treated with RLT were reported by Severi et al. [58], who treated 46 patients reaching a comprehensive DCR of 80%. Interestingly, 34 patients treated with [^177^Lu]Lu-DOTATATE obtained a longer median OS in comparison to those treated with [^90^Y]Y-DOTATOC (143 vs. 92 months, respectively). According to the authors, this result may be related both to DOTATATE’s higher affinity for SSTR2 (which is the type most overexpressed in PPGLs) and to the longer half-life of ^177^Lu and, consequently, prolonged residence time within the tumor lesions. Moreover, this study reports that sympathetic functioning PPGLs were associated to a shorter median PFS compared to non-functioning PPGLs. Prado-Wohlwend et al. [40] treated nine patients with [^177^Lu]Lu-DOTATATE and eight with [^131^I]MIBG, obtaining a PFS of 29 and 18.5 months and a DCR of 88.8% and 62.5%, respectively, for each therapy. Despite the low number of patients involved, a trend for a longer PFS was found for adrenal primary PPGLs treated with [^177^Lu]Lu-DOTATATE. Nevertheless, the authors suggest performing both [^68^Ga]Ga-DOTA-SSA PET/CT and [^123^I]MIBG SPECT/CT in each patient in order to offer a personalized theranostic approach based on the highest uptake intensity in one of the two imaging scans. This is consistent with the results by Jaiswal et al. [57] who found that a baseline SUVmax > 21 at [^68^Ga]Ga-DOTA-SSA PET/CT is a very strong predictor of response to [^177^Lu]Lu-DOTATATE (*p* < 0.0001).

[^117^Lu]Lu-DOTATATE treatment was safe and well-tolerated in the vast majority of patients treated, with only mild and transient side effects reported [40,41,53,55,56,57,58]. Of note, a patient presented a reactive painful swelling of tumor metastases that required escalation of analgesia [55]. In a very few cases, treatment was prematurely terminated due to hematologic toxicity (thrombocytopenia and anemia) [53]. Catecholamine crisis is an early infrequent complication in symptomatic PPGLs treated with [^177^Lu]Lu-DOTATATE. A combined α/β-adrenergic blockade should be considered when treating these patients, with a protocol similar to that used before adrenal surgery [59].

A special mention should be made for neuroblastoma, which is an aggressive tumor mainly affecting pediatric patients, that can overexpress SSTR [60,61]. However, there are still few studies conducted, and they are on a small number of patients. In particular, Fathpour et al. [62] treated five pediatric patients who were affected by relapsed or refractory metastatic neuroblastoma with [^177^Lu]Lu-DOTATATE. As a result, the authors report two complete responses, one partial response, and two progressive diseases, with an OS of 14.5 months. Hopefully, a pair of ongoing clinical trials (discussed below) aiming to evaluate intensified RLT in these aggressive tumors will provide more favorable results.

### 3.4. Meningiomas, CUP-NETs, and Other Rare Tumors Overexpressing SSTR

Meningiomas are the most common primary intracranial tumors, originating from arachnoid cap cells [63]. In most cases, meningiomas are single and benign, but occasionally, they can occur in multiple forms both in the brain and in the spinal cord. Despite being generally slow-growing, meningiomas can become very large, and the consequent mass effect can determine disability and become life-threatening. The WHO classifies meningiomas in three different grades of dedifferentiation that are associated with very different outcomes: from grade I (benign) to grade II (atypical) and III (anaplastic or malignant) [63]. In high-risk or symptomatic meningiomas, first choice treatment is usually surgery plus/or radiation therapy. The use of systemic treatments is not standardized, and the most promising results have been obtained with antiangiogenic treatments and mTOR inhibitors [64].

Meningiomas usually overexpress SSTRs. In particular, SSTR2 are usually overexpressed among skull base meningiomas. Conversely, spinal meningiomas usually express other SSTRs, while WHO grade III meningiomas are often SSTR-negative due to dedifferentiation and consequent loss of SSTR expression [65]. Overexpression of SSTR2 potentially allows RLT with [^177^Lu]Lu-DOTATATE, although this treatment is not currently approved for meningiomas due to the lack of large-scale randomized trials [66]. Nevertheless, in the future, RLT could hopefully carve out a role in recurrent or in unresectable meningiomas after other standard therapies have failed.

A few studies evaluated the efficacy of RLT in meningiomas, administrating 2–5 cycles of [^90^Y]Y-DOTATATE/-DOTATOC or [^177^Lu]Lu-DOTATATE/-DOTATOC [67,68]. In a meta-analysis performed by Mirian et al. [69], RLT—offered as mono-therapy or in combination with other oncological treatments—allowed a comprehensive DCR of 63% in refractory meningiomas. The 6-month PFS rates were 94%, 48%, and 0% for patients with WHO grade I, II, and III meningiomas, respectively, whereas the corresponding 1-year OS rates were 88%, 71%, and 52%, respectively. In a study by Seystahl et al. [70] RLT, offered mainly with [^177^Lu]Lu-DOTATATE (85% of patients), obtained 6-month PFS rates of 100%, 57%, and 0% for grade I, II, and III refractory meningiomas, respectively. In a recent study [68] on a small group of selected patients affected by WHO grade II refractory meningiomas, 6-month PFS was 85.7% and 1-year PFS was 66.7%. The treatment was safe and well tolerated.

Several studies confirmed the poor outcome for high grade meningiomas treated with RLT [68,71]. Conversely, Minczeles et al. [72] documented both a decline in tumor growth rate and a relatively good disease control in 15 patients who received [^177^Lu]Lu-DOTATATE, with low systemic toxicity. Probably, baseline SSTR2 expression may play a key role in the correct selection of patients with aggressive meningiomas to address to RLT.

An increase in treatment efficacy may be achieved via intra-arterial RLT injection, providing a significant tracer accumulation and promising improvement for the salvage treatment of meningioma patients [73,74]. Moreover, aggressive meningiomas may benefit of the combination of RLT with external beam radiotherapy [75,76], although further studies are necessary to determine its efficacy and survival improvements.

A special mention should be made for CUP-NETs, definable as metastatic NETs diagnosed from the histopathological assessment of a metastasis. CUP-NETs are not that infrequent, representing 20–25% of all diagnosed NETs. These tumors might originate from very small lesions, probably mainly arising from the GEP-NET district, and the absence of a known primary site is a negative prognostic factor, as it limits available therapeutic options [77]. Indeed, [^68^Ga]Ga-DOTA-SSA PET/CT is estimated to detect the unknown primary tumor only in about 60% of CUP-NET patients and this is an important limit to overcome, as CUP-NETs—despite usually intensively overexpressing SSTRs—are still “Lutathera^®^ orphans” [78,79].

Unfortunately, the literature lacks specific trials assessing RLT efficacy and safety on CUP-NETs, and the only available literature evidence is provided by mixed trials also including a few patients with SSTR-positive CUP-NETs [36,56,80,81]. In a large cohort of patients treated with [^177^Lu]Lu-DOTATATE, Brabander et al. [36] reported of 82 CUP-NET patients. Overall, DCR was reached in 78% of patients and median PFS and OS were 29 and 53 months, respectively. These results show that RLT may be effective in CUP-NETs patients, with a response rate intermediate between that obtained in GEP-NETs—who showed longer median OS (60 vs. 53 months, respectively)—and BCs—who showed shorter median PFS (20 vs. 29 months, respectively). These results are consistent with those reported by Demirci et al. [56] who treated 19 CUP-NETs, with an overall DCR of 84.2% and mean PFS and OS of 40 and 48 months, respectively. Once again, RLT outcomes in CUP-NETs were intermediate between those in GEP-NETs and BCs. In a large mixed cohort, Baum et al. [82] treated 151 CUP-NETs (mostly with [^177^Lu]Lu-DOTATATE, although a small percentage of patients was treated with [^90^Y]Y-DOTATATE/DOTATOC in the study), obtaining median PFS and OS of 13 and 46 months, respectively. CUP-NETS—together with BCs—were associated with a significantly shorter PFS at multivariate analysis if compared to pancreatic NETs despite showing a longer OS (50 vs. 43 months, respectively). Future prospective trials assessing efficacy and safety of RLT in CUP-NETs are desirable.

Potential use of [^177^Lu]Lu-DOTATATE in rare tumors is currently minimally explored, and there are only a few studies on restricted groups of patients. Moreover, several case reports tested [^177^Lu]Lu-DOTATATE for other malignancies, such as recurrent skull base phosphaturic mesenchymal tumor [83], mantle cell lymphoma [84], radioiodine refractory thyroid cancer [85], sarcoma [86], and breast cancer [87].

Medullary thyroid carcinoma (MTC) represents 1–5% of all thyroid malignancies, and it usually overexpress SSTR [88]. A few authors reported MTC patients treated with RLT, and a recent review reports biochemical and objective responses in 37.2% and 10.6% patients, respectively [89,90]. Maclean et al. [91] evaluated three patients treated with [^177^Lu]Lu-DOTATATE for aggressive atypical pituitary adenoma/carcinoma. They obtained mixed results and indicated that RLT with [^177^Lu]Lu-DOTATATE could be suitable for patients with good performance status and slowly progressive disease. Hasan et al. [92] tested the efficacy of RLT with [^177^Lu]Lu-DOTATATE on seven patients affected by esthesioneuroblastoma and achieved favorable clinical and imaging responses (median PFS and OS of 17 and 32 months, respectively). Treatment was well tolerated. Finally Adnan et al. [93] described RLT in two patients affected by primary soft tissue NET. In these two patients, they achieved both anatomic and metabolic responses after four cycles of [^177^Lu]Lu-DOTATATE.

The most relevant papers regarding RLT in “Lutathera^®^ Orphan” neoplasms are reported in Table 1.

### 3.5. RLT Retreatment

NETTER-1 clinical study has represented the milestone for approving RLT with Lutathera^®^ in patients with progressive and unresectable midgut, well-differentiated (G1, G2) NET with positive SSRT imaging. Indeed, patients group treated with Lutathera^®^ had a longer survival, expressed as PFS and OS, compared to those treated with only high-dose octreotide [5]. Nevertheless, despite these encouraging results, most patients relapse after RLT within a variable interval of time, leaving few options for future therapy. Therefore, retreatment with RLT, the so called “salvage RLT”, might represent a new valuable strategy for patients who present disease progression, although it is not currently approved. Until now, few clinical studies have been published [94,95,96,97,98,99,100,101,102] or are under investigation (NCT04954820, expected completion in 2029). All the studies, although conducted on small cohort of patients, have demonstrated an acceptable safety profile with a limited number of grade 3–4 toxicities, comparable to first-course RLT. Moreover, patients who underwent salvage RLT showed prolonged survival in terms of both PFS and OS. Recently, one of the largest clinical trials (involving 168 GEP-NET and 181 BCs) with the longest follow-up (80.8 months) showed a PFS of 14.6 months after re-treatment with two additional cycles of [^177^Lu]Lu-DOTATATE. In addition, OS was significantly longer in patients with BCs, GEP-NET, and midgut NET treated with salvage RLT compared to those in the nonrandomized control group. It is of note that adverse events were comparable between the initial four cycles of RLT and the salvage RLT group [94]. Similarly, van der Zwan et al. [100] demonstrated the efficacy and safety of retreatment in BCs patients, obtaining a significant longer OS in patients treated with salvage PRRT than in the control group (80.8 vs. 51.4 months). No grade III/IV renal toxicity occurred after retreatment, with mild and transient short-term side effects reported. Infrequent severe long-term toxicities comprehended acute leukemia or myelodisplasy but occurred only in 2% of patients. Furthermore, Sitani et al. [101] reported similar encouraging results in terms of the efficacy, survival, and toxicity of salvage RLT using the indigenous “direct route” [^177^Lu]Lu-DOTATATE, which is a cost-effective procedure for producing ^177^Lu adopted in most RLT centers in India.

Nevertheless, from the above mentioned studies emerge some questions that should be clarified in order to establish new recommendations for patients with progressive NET and potential candidates for salvage RLT [103,104]. Indeed, there is a wide variability in terms of cumulative administered activities, ranging between 14.8 and 63.8 GBq, as well as for the radioisotope used for salvage RLT. Although most of the studies have included only [^177^Lu]Lu-DOTATATE, Vaughan et al. [98] evaluated 45 patients treated with both [^90^Y]Y-DOTATATE and [^177^Lu]Lu-DOTATATE (45 and 2 at RLT1 and 29 and 18 at salvage RLT, respectively). Median PFS after salvage RLT was 17.5 months (range 11–23.8 months) with shorter PFS in males and patients with a high liver tumor burden. Again, salvage RLT was safe, with only one case of grade 4 renal toxicity. On the other hand, Severi et al. [96] assessed the role of RLT retreatment with [^177^Lu]Lu-DOTATATE in 26 GEP-NET patients who were previously treated with [^90^Y]Y-DOTATATE. The study showed an 84.6% DCR for salvage RLT, and PFS was similar to that of primary RLT (22 vs. 28 months, respectively). Moreover, low incidence of severe toxicities after salvage RLT was reported, with hemoglobin levels restored within 3 months after treatment and only one patient with persistent renal damage.

Another aspect that should be standardized regards the number of additional cycles, which is between 1 and 13. Recently, a prospective, randomized, phase II clinical trial was proposed, aiming to compare four versus two Lutathera^®^ retreatment cycles in patients with new progressions of a well-differentiated intestinal NET [102].

The answer to these open questions could be provided by personalized dosimetry [105]. Notably, Garske-Romàn et al. [106] performed salvage RLT with variable cycles (3–9) until reaching 23 Gy of absorbed dose (AD) to the kidneys unless the treatment had to be stopped due to other reasons. Remarkably, patients reaching the AD threshold to the kidneys (61.5%) showed longer median PFS (33 vs. 15 months) and OS (54 vs. 25 months) than those who did not. Despite reaching 23 Gy of AD to the kidneys, only 4% of patients demonstrated G2 kidney toxicity, while only one patient (0.5%) had G4 kidney toxicity. These results are consistent with those of Sundlöv et al. [107], who also reported that individualized RLT, tailored on renal dosimetry, seems feasible and safe without grade 3–4 toxicity being observed. These results hint that salvage RLT is safe and effective, particularly if offered with a tailored dosimetric approach. Moreover, this approach leads to an increased number of cycles in the majority of patients, thus providing a potential advantage in terms of outcomes.

The identification of other prognostic biomarkers is another open challenge for patients with NET undergoing salvage RLT. Galler et al. [81] analyzed whether two clinical variables associated with PFS during primary RLT were also prognostic parameters for salvage RLT. At multivariate analysis, only the higher size of the largest lesion was able to predict PFS, whereas aspartate aminotransferase/alanine aminotransferase ratio (named “De Ritis ratio”) was not.

### 3.6. Ongoing Clinical Trials

Overall, 74 trials were retrieved from the research on https://clinicaltrials.gov/ (accessed on 13 February 2023). Thirty-nine trials met the inclusion criteria defined for this review, and their details were included in Table 2.

Among those, 2 trials were withdrawn and 2 are already concluded, with the remaining 35 currently ongoing. Interestingly, most trials include meningiomas or other infrequent tumors overexpressing SSTR (n = 16) and non-GEP NET (n = 7). Moreover, the evaluation of RLT outcomes in PPGLs is the main aim of six trials. Notably, seven trials are recruiting also pediatric patients, who are currently excluded from Lutathera^®^ indications. Among those, two trials are designed to investigate an intensified dosing schedule in pediatric patients affected by neuroblastoma. Indeed, trials investigating [^177^Lu]Lu-DOTATATE in neuroblastoma patients failed so far, and this can be due both to the insufficient activities administered and to the too long intervals between the cycles of therapy [109]. Therefore, following the example of [^131^I]MIBG, current trials are evaluating intensified cycles for this poor prognosis rapidly proliferating tumor. One trial is assessing RLT as neoadjuvant treatment in pancreatic NET, which is another promising future indication of [^177^Lu]Lu-DOTATATE [12]. Finally, eight trials involve NET G3, five BCs, and one trial aims to assess efficacy and safety of salvage RLT.

## 4. Discussion

Lutathera^®^ approval represented a milestone in the history of NET management. Indeed, these infrequent and very heterogeneous neoplasms have always represented a challenge for clinicians, as patients may present at diagnosis with very high burden of disease, poorly responsive to conventional therapies [114]. Therefore, allowing access to RLT to the largest possible population may offer an additional weapon to improve NET patients’ outcomes. Nevertheless, only a limited portion of NET patients are currently eligible for RLT outside clinical trials.

G3 GEP-NET tumors with Ki-67 index < 55% currently have very few therapeutic options but may be among the first “orphans” to gain access to Lutathera^®^ thanks to the NETTER-2 trial [18]. Probably, these neoplasms, which present a moderate differentiation and a high Ki-67 index, may benefit from combined RLT and chemotherapy (i.e., capecitabine and/or temozolomide), with the latter working both as radiosensitizer agent and offering a therapeutic effect on [^18^F]FDG-positive lesions [115]. These approaches could be useful also in treating aggressive meningiomas, mainly grade II. Even though the literature is limited in describing therapy for these tumors, available data suggest that RLT, alone or combined with systemic drugs, might be taken into account based on meningiomas’ SSTR expression at baseline imaging. Similarly, the literature results indicate that RLT proved to be promising in prolonging survival and delaying disease progression in BCs, which are already treated with off-label RLT in some countries. However, the optimal timing of RLT in the therapeutic management of BCs is still debated and prospective trials are needed to strengthen current preliminary evidence.

PPGLs represent another group of “Lutathera^®^ orphans”. Despite quite promising literature evidence in terms of safety and efficacy of RLT, further prospective trials are still needed to reach treatment approval [116]. A high baseline uptake at [^68^Ga]Ga-DOTA-SSA PET/CT is reported as a strong predictor of RLT efficacy in these neoplasms, that currently present very poor therapeutic options available [57]. Interestingly, RLT seems to be particularly useful to improve the quality of life in patients with catecholamine secretion, allowing to reduce anti-hypertensive drugs [41]. Overall, a DCR between 70–80% is reported for these neoplasms treated with RLT, which is higher than that reached by [^131^I]MIBG and with a safer toxicity profile [40,41]. Notably, the treatment of patients with catecholamine secretion must be handled gingerly due to the infrequent but possible therapy-induced catecholamine crisis. Therefore, this treatment could have a future limited to a small number of selected RLT referral centers capable of managing this kind of emergency.

Finally, literature evidence reports that retreatment with RLT is safe and effective and will hopefully obtain approval in the upcoming years [94,100]. NET patients usually have long life expectancy, and they often relapse after treatment [117]. Therefore, the possibility of offering a second-course RLT would be of great clinical usefulness. Future trials should focus on obtaining a joint standardized therapeutic program for salvage RLT both in terms of number of cycles and activity to inject per cycle. Dosimetry is expected to play a key role for this “orphan indication”, helping to improve personalization and maximizing the possible treatment benefits, albeit helping to maintain safe AD to the organs at risk.

## Figures and Tables

**Table 1:** Response rate and outcomes in the most relevant papers on neuroendocrine “orphan” neoplasms treated with [^177^Lu]Lu- RLT.

Type of Disease	Authors, Reference	N of pts	[^177^Lu]Lu-DOTATATE (n of Cycles)	Phase Study	ORR	DCR	Median (Range) FUP Time	Median PFS	Median OS
Bronchial Carcinoids	Ianniello et al. [32]	34	4–5	II	–	80%	29 (7–69) mo.	TC: 20.1 (11.8–26.8) mo. AC: 15.7 (10.6–25.9) mo.	48.6 (26–nr) mo.
	Van Essen et al. [34]	9	4	Pilot	56%	89%	–	–	–
	Lim et al. [35]	48	median 4	RA	33%	83%	33 mo.	–	49 (3–91) mo.
	Brabander et al. [36]	443	cumulative dose: 27.8–29.6 GBq	RA	39%	60%	–	29 mo.	63 mo.
	Mariniello et al. [39]	114	4–6	RA	29.2%	75%	–	28 mo.	58.8 mo.
PPGL and PHEO	Van Essen, [53]	12	4	Pilot	18%	73%	13 (4–30) mo.	–	–
	Kong et al. [41]	20	4	RA	36%	86%	28 (5–74) mo.	39 mo.	28 mo.
	Severi et al. [58]	46	4–5	PA	–	80.4%	73 (5–146) mo.	nr	142.6 (103.1–146.2) mo.
	Prado-Wohlwend et al. [40]	9	4	Pilot	–	88.8%	–	29 mo.	–
Meningiomas	Salgues et al. [68]	8	4	RA	–	–	–	6 mo PFS = 85.7% (WHO II)	–
	Seystahl et al. [70]	20	median 3	RA	–	50% (stable disease)	–	32.2 (WHO I); 7.6 (WHO II); 2.1 (WHO III) mo.	Nr (WHO I and II); 17.2 mo. (WHO III meningiomas)
	Minczeles et al. [72]	15	4	RA	40%	40%	13 (10–27) mo.	7.8 (5.3–10.3) mo.	13.6 (10.3–17.0) mo.
CUP-NET	Brabander et al. [36]	82	4	RA	35%	78%	–	29 mo.	53 mo.
	Demirci et al. [56]	19	median 6	RA	36.8%	84.2%	30.6 mo.	40.9 (4.9–31.2) mo.	48.3 (4.5–39.5) mo.
	Baum et al. [82]	1048	4	RA	–	–	–	13 (9.5–16.4) mo.	53 (37.5–68.4) mo.

ORR = overall response rate; DCR = disease control rate (complete, partial, and stable disease); mo.= months; nr = not reached; RA = retrospective analysis; PA = prospective analysis.

**Table 2:** Ongoing clinical trials assessing [^177^Lu]Lu-DOTATATE RLT in “Lutathera^®^ Orphans”. The short title shown in the table; to facilitate any consultation, this is the original title entered by the experimenters on the site and not modified, even where not formally correct.

S/E	NCT Number and Brief Title	Phase	Country	Conditions	Aim of Study	Orphan Condition
October 2021 / September 2029	NCT04954820 [102] Assessment of the Schema of Retreatment With Lutathera^®^ in Patients With New Progression of Intestinal Well- differenciated NET.	Phase II	France	NET; intestinal well differentiated endocrine tumor; progressive disease.	To evaluate the efficacy of 2 additional cycles of Lutathera^®^ vs. active surveillance in patients already retreated with two cycles Lutathera^®^ for a new progression of intestinal NET.	Retreatment
April 2022 / December 2025	NCT05178693 Lutathera and ASTX727 in Neuroendocrine Tumours.	Phase I	United Kingdom	NETs G1 or G2 or G3	To determine whether pretreatment with ASTX727 before Lutathera^®^ results in re-expression of SSTR2 in patients with metastatic NETs.	NET G3
April 2023 / July 2024	NCT05687123 Testing the Addition of Sunitinib Malate to Lutetium Lu 177 Dotatate (Lutathera) in Pancreatic Neuroendocrine Tumors.	Phase I	United States of America	PAN-NET of any grade	To test the safety, side effects, and best dose of sunitinib malate in combination with Lutathera^®^ in treating patients with PanNETs.	NET G3
January 2020 / July 2027	NCT03972488 Study to Evaluate the Efficacy and Safety of Lutathera in Patients With Grade 2 and Grade 3 Advanced GEP-NET.	Phase III	International	GEP-NET	To compare Lutathera^®^ plus long-acting octreotide vs. high dose long-acting octreotide as first line treatment in G2 and G3 GEP-NET.	NET G3
June 2020 / September 2024	NCT04525638 A Clinical Study to Assess the Combination of Two Drugs (^177^Lu-DOTATATE and Nivolumab) in Neuroendocrine Tumours.	Phase II	Spain	NETNEC	To evaluate the efficacy and safety of [^177^Lu]Lu-DOTATATE in combination with nivolumab in adult patients with G3 NETs or NECs.	NET G3 and NEC
March 2022 / March 2028	NCT05247905 Comparing Capecitabine and Temozolomide in Combination to Lutetium Lu 177 Dotatate in Patients With Advanced Pancreatic Neuroendocrine Tumors.	Phase II	United States of America	PAN-NET G1 or G2 or G3	To find out whether capecitabine and temozolomide or [^177^Lu]Lu-DOTATATE may kill more tumor cells in patients treated for metastatics or unresectable PanNETs.	NET G3
August 2018 / December 2025	NCT03457948 Pembrolizumab With Liver-Directed or Peptide Receptor Radionuclide Therapy for Neuroendocrine Tumors and Liver Metastases.	Phase II	United States of America	NET G1 or G2 or G3 of any primary site, including unknown primary site	To study the effectiveness of pembrolizumab and liver-directed therapy or Lutathera^®^ in treatment of patients with symptomatic and/or progressive NETs with liver metastases.	NET G3—NET of any, primary origin, including CUP-NETs
November 2022 / November 2027	NCT05278208 Lutathera for Treatment of Recurrent or Progressive High- Grade CNS Tumors or Meningiomas Expressing SST2A.	Phase I e II	United States of America	High-grade CNS primary central nervous system neoplasm; meningioma;	To evaluate safety and efficacy of Lutathera^®^ in pediatric and young adult patients with progressive or recurrent high-grade CNS tumors and meningiomas (any grade) that express SSTR2.	Meningiomas and other tumors overexpressing SSTR— pediatric patiens
August 2022 / August 2028	NCT04711135 Study to Evaluate Safety and Dosimetry of Lutathera in Adolescent Patients With GEP-NETs and PPGLs.	Phase II	International	GEP-NET G1 or G2 PPGLs	To evaluate safety and dosimetry of Lutathera^®^ in adolescent patients with SSTRs positive GEP-NETs and PPGLs.	PPGLs—pediatric patiens
August 2020 / June 2021	NCT03923257 Withdrawn, competing clinical trial opening Dosimetry Guided PRRT With ^177^Lu-DOTATATE in Children and Adolescents.	Phase I e II	United States of America	NET PPGLs	Assess Lutathera^®^ in children and adolescents with neuroendocrine tumors and PHEO or PGL.	PPGLs—pediatric patiens
January 2023 / January 2025	NCT03966651 A Clinical Study Evaluating the Safety of Peptide Receptor Radionuclide Therapy (PRRT) With ^177^Lu-DOTA0-Tyr3- Octreotate in Children With Refractory or Recurrent Neuroblastoma Expressing Somatostatin Receptors.	Phase I	France	Neuroblastoma	To define the Maximum Tolerated Dose (MTD) of [^177^Lu]Lu-DOTATATE in children with refractory or recurrent neuroblastoma.	Neuroblastoma—Pediatric patiens
July 2014 / December 2023	NCT02236910 [108] An Open Label Registry Study of Lutetium-177 (DOTA0, TYR3) Octreotate (Lu-DOTA-TATE) Treatment in Patients With Somatostatin Receptor Positive Tumours.	Phase II	Canada	NET any primary origin (male or female ≥ 14–90 years of Age)	To assess the efficacy (PFS and OS), safety and quality of life of patients treated with [^177^Lu]Lu-DOTATATE.	Pediatric patiens—NET of any primary origin
April 2014 / December 2033	NCT01876771 A Trial to Assess the Safety and Effectiveness of Lutetium-177 Octreotate Therapy in Neuroendocrine Tumours.	Phase II	Canada	NET any primary origin (male or female ≥ 14–90 years of age)	To assess efficacy and safety of [^177^Lu]Lu-DOTATATE treatment in patients with SSTR positive tumors and the assess the effect on quality of life and survival.	Pediatric patiens—NET of any primary origin
May 2021 / May 2031	NCT04903899 [109] ^177^Lutetium-DOTATATE in Children With Primary Refractory or Relapsed High-risk Neuroblastoma	Phase II	Sweden	Neuroblastoma	Assess an intensified dosing schedule to deliver 2 doses over a 2-week period in neuroblastoma	Neuroblastoma—Pediatric patiens
May 2019 / May 2025	NCT03971461 Phase II Study of ^177^Lu-DOTATATE Radionuclide in Adults With Progressive or High-risk Meningioma.	Phase II	United States of America	Meningioma	To evaluate the efficacy of Lutathera^®^ in patients with progressive WHO I-III or residual high-risk SSTR-positive meningioma.	Meningiomas and other tumors overexpressing SSTR
December 2023 / October 2025	NCT04614766 A Clinical Trial Evaluating the Safety of Combining Lutathera(R) and Azedra(R) to Treat Mid-gut Neuroendocrine Tumors	Phase I e II	United States of America	NET midgut G1 or G2 PPGLs	To identify the best tolerated doses of Lutathera^®^ and Azedra^®^ when coadministered to treat NETs.	PPGLs
November 2017 / Augus 2020	NCT03325816 [110] Nivolumab and ^177^Lu-DOTA0-Tyr3-Octreotate for Patients With Extensive-Stage Small Cell Lung Cancer	Phase I e II completed	United States of America	Small cell lung cancer	To assess safety and tolerability of combined treatment with Lutathera^®^ and nivolumab, in subjects with SCLC or advanced or inoperable lung NETs and also to determine if PD-L1 expression increases the chances of a response	Meningiomas and other tumors overexpressing SSTR
October 2020 / November 2024	NCT04039516 Carcinoid Heart Disease and Peptide Receptor Radiotargetted Therapy	Phase II	United Kingdom	GEP-NET G1 or G2 or Lung NET/bronchial carcinoid	To assess progression of carcinoid heart disease in patients treated with Lutathera^®^ compared to best supportive care.	Bronchial carcinoid
September 2020 / September 2024	NCT04544098 Lutathera in People With Gastroenteropancreatic (GEP), Bronchial or Unknown Primary Neuroendocrine Tumors That Have Spread to the Liver	Phase I	United States of America	GEP-NET or CUP- NET G1 or G2 or G3 typical or atypical lung/bronchial carcinoid	To investigate intrahepatic arterial and intravenous infusion of Lutathera^®^ in patients with liver-dominant metastatic GEP-NETs, bronchial NETs, or well-differentiated CUP-NETs.	NET G3—CUP-NETs—Bronchial carcinoid
March 2020 / September 2024	NCT04082520 Lutathera for the Treatment of Inoperable, Progressive Meningioma After External Beam Radiation Therapy	Phase II	United States of America	Meningioma	To assess Lutathera^®^ in treating patients with unresectable meningioma or progressive meningioma after EBRT.	Meningiomas and other tumors overexpressing SSTR
September 2021 / July 2024	NCT04665739 Testing Lutetium Lu 177 Dotatate in Patients With Somatostatin Receptor Positive Advanced Bronchial Neuroendocrine Tumors.	Phase II	United States of America	Bronchial carcinoid	To assess if Lutathera^®^ is more effective than everolimus in shrinking or stabilizing advanced bronchial neuroendocrine tumors.	Bronchial carcinoid
June 2019 / September 2020	NCT04106843 Radioactive Drug (^177^Lu-DOTATATE) for the Treatment of Locally Advanced, Metastatic, or Unresectable Rare Endocrine Cancers	Phase II withdrawn, no participants registered.	United States of America	PPGLs, Parathyroid carcinoma, pituitary gland carcinoma; thyroid gland medullary carcinoma	To assess effectiveness of Lutathera^®^ in patients with infrequent locally advanced or unresectable or metastatic endocrine cancers.	PPGLs and meningiomas and other tumors overexpressing SSTR
May 2022 / June 2025	NCT05109728 A Dose Finding Study of [^177^Lu]Lu-DOTA-TATE in Newly Diagnosed Glioblastoma in Combination With Standard of Care and in Recurrent Glioblastoma as a Single Agent.	Phase I	France, Portugal, Spain, Switzerland	Glioblastoma	To establish the recommended dose of Lutathera^®^ in combination with the standard of care or as single agent in three different groups of participants with glioblastoma.	Meningiomas and other tumors overexpressing SSTR
April 2020 / June 2023	NCT04375267 [111] ^177^Lu-DOTA-TATE and Olaparib in Somatostatin Receptor Positive Tumours.	Phase I	Sweden	Advanced GEP-NETs (poor prognosis and Ki67 > 15%), thymomas, meningiomas, mesothelioma	Phase I study of Lutathera^®^ in combination with the PARP-inhibitor olaparib for treatment of patients with SSTRs positive tumors.	Meningiomas and other tumors overexpressing SSTR
March 2020 / December 2022	NCT04385992 Neoadjuvant PRRT With ^177^Lu-DOTATATE Followed by Surgery for Resectable PanNET	Phase II	Italy	PAN-NET	To evaluate safety and efficacy of neoadjuvant PRRT with [^177^Lu]Lu-DOTATATE followed by surgical resection for resectable nonfunctioning PanNETs at high risk of recurrence.	Neoadjuvant purpose
September 2022 / December 2022	NCT04529044 ^177^Lu-DOTATATE for the Treatment of Stage IV or Recurrent Breast Cancer.	Phase II	United States of America	Breast cancer	To investigate the efficacy of Lutathera^®^ in treating patients with stage IV or recurrent breast cancer positive for SSTR2.	Meningiomas and other tumors overexpressing SSTR
October 2011 / November 2018	NCT01456078 [112] A Multicenter Study Evaluating Efficacy and Safety of ^177^Lu-DOTA-TATE Based on Kidney-Dosimetry in Patients With Disseminated Neuroendocrine Tumors.	Phase II completed	Sweden	NET Liver metastases	To optimize and personalize [^177^Lu]Lu-DOTATATE treatment in patients with metastatic NETs by performing renal dosimetry and to determine the biological effective dose for renal toxicity.	NET of any primary origin
July 2022 / November 2024	NCT05142696 A Safety Study of [^177^Lu]Lu-DOTA-TATE in Newly Diagnosed Extensive Stage Small Cell Lung Cancer (ES-SCLC) Patients in Combination With Carboplatin, Etoposide and Tislelizumab.	Phase I	France, Spain	Small cell lung cancer	To establish a safe and well tolerated dose of Lutathera^®^ in combination with carboplatin, etoposide, and tislelizumab in induction treatment and with tislelizumab in maintenance treatment in newly diagnosed patients with extensive stage SCLC.	Meningiomas and other tumors overexpressing SSTR
May 2021 / December 2035	NCT04949282 Spanish Series of Patients Treated With the Radionuclide Lutetium177	Observational study	Spain	NET of any primary origin; other tumors overexpressing SSTR	To assess the state of art of Lutathera^®^ treatment in Spain in terms of efficacy, tolerance, and safety in routine clinical practice in different types of tumors.	NET of any primary origin—meningiomas and other tumors overexpressing SSTR
January 2023 / June 2024	NCT05198479 Phase II ^177^Lu-DOTATATE Study in Metastatic NPC With a Safety Run-in	Phase II	Singapore	Metastatic nasopharyngeal cancer	To assess 6-month PFS in metastatic SSTR positive NPC treated with Lutathera^®^ after progression to 2 or more lines of therapy or exhausted standard therapy	Meningiomas and other tumors overexpressing SSTR
February 2023 / February 2025	NCT05583708 Phase II Study of Peptide Receptor Radionuclide Therapy in Combination With Immunotherapy for Patients With Merkel Cell Cancer.	Phase II	United States of America	Merkel cell carcinoma	A single-arm study with safety run-in of RLT with Lutathera^®^ in bombination with immunotherapy for patients with Merkel cell cancer.	Meningiomas and other tumors overexpressing SSTR
October 2017 / 1 January 2026	NCT03206060 Lu-177-DOTATATE (Lutathera) in Therapy of Inoperable Pheochromocytoma/Paraganglioma.	Phase II	United States of America	PHEO Paraganglioma	To evaluate the safety and tolerability of Lutathera^®^ in unresectable SSTR positive tumors.	PPGLs
November 2004 / January 2021	NCT04029428 Peptide Receptor Radionuclide Therapy in the Treatment of Advanced, Non-resectable and/or Symptomatic Tumors With SSTR Overexpression.	Phase II	Poland	GEP-NET (G1, G2 and G3), bronchial–pulmonary carcinoids (BPCs atypical-AC or typical-TC), PPGLs, CUP-NET	To evaluate RLT with [^90^Y]Y-DOTATATE, [^177^Lu]Lu-DOTATATE, or combined.	PPGLs—bronchial carcinoids—CUP-NETs—NET G3
May 2016 / May 2021	NCT03454763 Optimizing the Interval Between Cycles of PRRT With ^177^Lu-DOTATATE in sstr2 Positive Tumors.	Phase II	Italy	NET of any primary origin; other tumors overexpressing SSTR	To optimize the interval between cycles of PRRT with [^177^Lu]Lu-DOTATATE in SSTR2-positive tumors	NET of any primary-origin meningiomas and other tumors overexpressing SSTR
October 2010 / August 2015	NCT01237457 ^177^Lutetium-DOTA-Octreotate Therapy in Somatostatin Receptor-Expressing Neuroendocrine Neoplasms.	Phase II	United States of America	Pheochromocytoma; bronchial carcinoids; other tumors overexpressing SSTR	This is a phase II treatment protocol offering [^177^Lu]Lu-DOTATATE therapy for SSTR positive cancers.	Bronchial carcinoids—meningiomas and other tumors overexpressing SSTR
December 2022 / December 2031	NCT05249114 Study of Cabozantinib With Lu-177 in Patients With Somatostatin Receptor 2 Positive Neuroendocrine Tumors.	Phase I	United Stated of America	fore-, mid-, or hindgut, including pancreas, or those with an unknown primary	To establish the MTD of cabozantinib in combination with Lutathera^®^ at a standard dose followed by continuation cabozantinib.	CUP-NETs
October 2020 / January 2024	NCT04261855 Targeted Therapy and Avelumab in Merkel Cell Carcinoma.	Phase I and Phase II	Australia	Metastatic Merkel cell carcinoma	To evaluate the safety and anti-tumor activity of Lutathera^®^ or EBRT in combination with avelumab in patients with mMCC.	Meningiomas and other tumors overexpressing SSTR
April 2016 / December 2026	NCT02754297 [105,113] Personalized PRRT of Neuroendocrine Tumors	Phase II	Canada	NET of any primary origin	To Evaluate personalized patient-tailored [^177^Lu]Lu-DOTATATE RLT.	NET of any primary origin
August 2013 / February 2017	NCT01915485 Radiolabeled Molecules for Medullary Thyroid Cancer	Phase IV	Brazil	Medullary thyroid cancer	To assess [^177^Lu]Lu-DOTATATE RLT in SSTR positive medullary Thyroid cancer.	Meningiomas and other tumors overexpressing SSTR

AC = atypical carcinoid; ASTX727 = oral decitabine and cedazuridine; BPC = bronchial–pulmonary carcinoids; CNS = central nervous system; CUP-NET = neuroendocrine tumor—cancer of unknown primary; EBRT = external beam radiation therapy; Ga = gallium; GEP-NET = gastroenteropancreatic neuroendocrine tumor; Lu = lutetium; mMCC = metastatic Merkel cell carcinoma; MRI = magnetic resonance imaging; MTD = maximum tolerated dose; NEC = neuroendocrine carcinoma; NET = neuroendocrine tumor; NPC = nasopharyngeal cancer; PAN-NET and PanNET = pancreatic neuroendocrine tumor; PARP = poly-ADP ribose polymerase; PET = positron emission tomography; PFS = progression-free survival; PHEO = pheochromocytoma; PPGLs = pheochromocytomas and paragangliomas; PI= principal investigator; PRRT = peptide receptor radionuclide therapy; QoL = quality of life; RECIST = response evaluation criteria in solid tumors; RLT = radioligand therapy; S/E= start/end study; SCLC = small cell lung cancer; SSA = somatostatine analogue; SSTR = somatostatine receptor; TC = typical carcinoid.

## Data Availability

Trials data cited can be consulted on the following site: https://clinicaltrials.gov/ (accessed on 13 February 2023).
